# Genetic and phenotypic variability of iris color in Buenos Aires population

**DOI:** 10.1590/1678-4685-GMB-2017-0175

**Published:** 2018

**Authors:** Diana María Hohl, Brenda Bezus, Julia Ratowiecki, Cecilia Inés Catanesi

**Affiliations:** 1 Laboratorio de Diversidad Genética, Instituto Multidisciplinario de Biología Celular IMBICE (CONICET-UNLP-CIC), La Plata, Buenos Aires, Argentina; 2 Facultad de Ciencias Exactas, Universidad Nacional de La Plata, La Plata, Buenos Aires, Argentina; 3 Centro de Estudios Médicos e Investigaciones Clínicas CEMIC CONICET, Buenos Aires, Argentina; 4 Facultad de Ciencias Naturales y Museo, Universidad Nacional de La Plata, La Plata, Buenos Aires, Argentina

**Keywords:** Population genetics, Iris color, forensic genetics, phenotype determination, Argentinian population

## Abstract

The aim of this work was to describe the phenotypic and genotypic variability related to iris color for the population of Buenos Aires province (Argentina), and to assess the usefulness of current methods of analysis for this country. We studied five Single Nucleotide Polymorphisms (SNPs) included in the IrisPlex kit, in 118 individuals, and we quantified eye color with Digital Iris Analysis Tool. The markers fit Hardy-Weinberg equilibrium for the whole sample, but not for rs12913832 within the group of brown eyes (LR=8.429; *p*=0.004). We found a remarkable association of *HERC2* rs12913832 GG with blue color (*p* < 0.01) but the other markers did not show any association with iris color. The results for the Buenos Aires population differ from those of other populations of the world for these polymorphisms (*p* < 0,01). The differences we found might respond to the admixed ethnic composition of Argentina; therefore, methods of analysis used in European populations should be carefully applied when studying the population of Argentina. These findings reaffirm the importance of this investigation in the Argentinian population for people identification based on iris color.

## Introduction

Iris color is a conspicuous phenotypic trait in humans, and its variation is one of the most relevant manifestations of the physical aspect among individuals. This phenotypic trait is polygenic, and its genetic background is variable across different populations. Eye color is subjected to certain changes along the lifetime (Sun *et al.*, 2014). However, this phenotype is mainly influenced by genetic factors acting together as a quantitative trait, giving rise to a range of colors from blue to dark brown (Brues, 1975) generated by complex genetic interactions (Kanetsky *et al.*, 2002). The importance of its study relies in its potential application in Forensic Genetics, as a tool for predicting Externally Visible Characteristics (Tully, 2007; Kayser and Schneider, 2009).

In recent years the principal genes involved in phenotype determination of iris color have been identified, including *HERC2, IRF4, SLC24A4, SLC45A2* and *TYR* among the most important ones*.* Within these genes, particular SNPs have been reported as responsible for the pigmentation variation, allowing the determination of iris color in European populations with small levels of error. These SNPs are included in the IrisPlex (Walsh *et al.*, 2011a) genotyping system for eye color prediction, which is currently being used in different countries for identification purposes. However, the available data has been collected mainly from European populations (Sulem *et al.*, 2007; Liu *et al.*, 2009; Mengel-From *et al.*, 2009; Kastelic and Drobnic, 2012), while the information about the variability of these SNPs in other populations of the world, and especially of Latin America, is yet incomplete (Beleza *et al,.* 2013; Edwards *et al.*, 2015).

Concerning phenotypic variation, until recently, the assignment of iris color was determined only by qualitative methods (Grigore and Avram, 2015). Nevertheless, the qualitative description of this phenotypic trait by assigning each eye subjectively to a group (blue, green or brown), could result in some bias in the classification. This bias usually hinders to reproduce the results, especially when the study attempts to relate these phenotypes with certain genetic markers that could be associated to minor variations in eye color. By using a quantitative method, the bias in determinations given by human subjectivity can be diminished, facilitating the comparison of studies on eye color genetics (Andersen *et al.*, 2013). At present, digital classification of iris color through software made possible a more objective quantification. Different programs were specifically designed for this purpose, such as the Digital Iris Analysis Tool (DIAT), developed by Jeppe Andersen (Andersen *et al.*, 2013), and a web application developed by David Cha (Edwards *et al.*, 2015). In particular, the DIAT software counts the number of blue and brown pixels in the iris image and calculates a Pixel Index of the Eye (PIE-score) that describes the eye color quantitatively. The PIE-score ranges from -1 to 1 (brown to blue, respectively). As the understanding of genes and markers responsible for intermediate iris color is still limited at present (Edwards *et al.*, 2015), the use of software for quantitative methods may improve the analysis.

In Argentina, immigrations and admixture played a fundamental role in the constitution of the current genetic composition of the population. Before the 16^th^ century, Native Americans were the original and only people living in the region. Between the 16^th^ and 18^th^ centuries a relatively small number of Spanish settlers arrived, and then African individuals were forced to migrate between the 17^th^ and 18^th^ centuries. Later, a great European immigration wave took place between 1850 and 1955, mainly composed of Spanish and Italians, and a smaller amount of Germans, Polish and Russians. Also, a small number of Jews and inhabitants from Asia, Middle and Near East, arrived (Devoto, 2007, Salas *et al.*, 2008). Consistently, Avena *et al.* (2012) reported an average autosomal ancestry of 65% European, 31% Native American and 4% African for Argentinians, although a gradient of different distribution of frequencies was found along the country. As a consequence, the genotypic features of Native Americans and Africans have diminished (Avena *et al.*, 2001), and the European traits of facial appearance, skin, eyes and hair color may predominate in some regions of Argentina (Merenson, 2015). According to official data (INDEC, 2010), from a total of approximately 40 million individuals in the country, 150,000 are Afro-descendant and approximately one million are Native or predominantly of Native ancestry. Besides, since the middle of 20^th^ century, immigration from neighboring South American countries, mainly Paraguay and Bolivia, increased, modifying the genetic composition of Argentinians, contributing more to the Amerindian genetic proportion (Avena *et al.*, 2006).

Populations of different ethnic and geographic origin could carry distinct allele variants of the above mentioned genes, with different effects on phenotype (Frudakis *et al.*, 2003; Sturm and Frudakis, 2004; Duffy *et al,.* 2007; Sulem *et al.*, 2007; Mengel-From *et al.*, 2009; Nan *et al,.* 2009; Walsh *et al.*, 2011a; Cerqueira *et al.*, 2012, 2014; Kastelic and Drobnic, 2012; Beleza *et al.*, 2013; Martinez-Cadenas *et al,.* 2013; Kastelic *et al.*, 2013; Lim and Oh, 2013; Ruiz *et al.*, 2013; Wilde *et al.*, 2014). In this way, the availability of the genetic information responsible for iris color determination in Argentina will provide new tools for its future application in individual identification. In our investigation, we studied a sample from Buenos Aires province in Argentina. The Buenos Aires province (Buenos Aires from now on) population is composed by individuals from all around the country, and they can be considered representative of the Argentinian population. The aim of this work was to describe the genetic component related to iris color variation in Argentina, and to assess the usefulness of current methods of analysis for a Latin American population, not only by checking the relationship between IrisPlex markers and eye color, but also by measuring the phenotypic variation quantitatively. Our results, the first for the country, showed genotype differentiation in the Buenos Aires population, thus contributing to phenotypic individual identification in forensic cases.

## Materials and Methods

### Sampling, DNA extraction, and phenotypic classification

We collected samples of 118 volunteer adult donors (69 females and 49 males) from Buenos Aires. This project was approved by the Ethics Committee for Biomedical Research at IMBICE, and donors provided individual written informed consent. The age of participants ranged between 18 and 50 years to avoid changes in iris color due to late age of individuals (Sun *et al.*, 2014). We obtained DNA from mouthwash through a standard technique using Proteinase K (Promega, USA) and LiCl (Gemmel and Akiyama, 1996), and we registered iris color variation with a digital camera (Nikon Coolpix s3400, 20 megapixels) using fill-flash. We avoided the variation of environmental light incidence and added a flashlight with a standardized constant light intensity. We were able to categorize 86 iris phenotypes out of the 118 individuals following Andersen *et al.* (2013), by using a custom designed software Digital Iris Analysis Tool (DIAT), gently provided by Dr. Jeppe D. Andersen (Dept. of Forensic Medicine, Faculty of Health and Medical Sciences, University of Copenhagen).

### Genotyping

We designed oligonucleotides for genotyping the SNPs rs12896399, rs1393350, rs12913832 and rs1689198, while those for rs12203592 were obtained from a previous publication (Walsh *et al.*, 2013). PCR reactions were performed using recombinant *Taq* polymerase (T-Plus from Inbio-Highway, Tandil, Argentina) in a thermocycler (MPI, La Plata, Argentina). For rs12203592, rs12896399 and rs1393350, we performed allele-specific PCR followed by electrophoretic separation in 1.8% agarose (Promega, USA) electrophoresis and staining with GelRed^TM^ (Biotium, USA). The SNPs rs12913832 and rs16891982 were genotyped by PCR-RFLP, using *Dde*I (New England Biolabs, USA) and *Hinf*I enzymes (Promega, USA) respectively for restriction digestion, followed by 8% polyacrylamide (Promega, USA) electrophoresis. Bands were recorded in a Gel Doc^TM^ XR+ system (Bio-Rad Laboratories, California, USA).

### Data analysis

We used data from the five SNPs included in this work for calculating allele and genotype frequencies, and for testing Hardy-Weinberg equilibrium (HWE) by comparison of observed and expected heterozygosity values through exact test (Markov chain) with Arlequin software 3.5 (Excoffier and Lischer, 2010). We also computed nucleotide diversity and *Fis* for the Buenos Aires population using the same software and determined the distribution of iris color variability as well. The mean, median and standard deviation for color and markers were calculated. We used a median regression analysis adding each SNPs as dummy variable to assess the association among genetic polymorphism. Additionally, a possible link between sex and iris color was verified, as proposed by Martinez-Cadenas *et al.* (2013) by Chi-square test with Yates correction.

*Fst* and AMOVA were analyzed with Arlequin 3.5 for comparison with previous literature data for Native Americans (3 countries: Columbia, Brazil, and Mexico, all native populations, total 61 individuals), Africans (6 native populations: Mbuti Pygmies, Biaka Pygmies, Mandenka, Yoruba, San, and Bantu, total 102 individuals), East Asians (3 countries: Japan, China and Russia (Siberia), total 230 individuals) and Europeans (4 countries: France, Italy, United Kingdom (Orkney Islands), and Russia, total 151 individuals) (Walsh *et al.*, 2011a). For *Fst,* a Bonferroni correction was used (Armstrong 2014). For AMOVA analysis, populations were grouped by continent, either including data from Africa, or comparing data more closely related by origin.

Population structure and ancestry estimation were done with Structure 2.3 (Pritchard *et al.*, 2000), assuming an admixture model, for K values between 2 and 5 (burn-in period: 10,000), and we compared Buenos Aires population to those above mentioned (Walsh *et al.*, 2011a) by non-metric multidimensional scaling (MDS) using the software package PAST (Hammer *et al.*, 2001).

## Results

**Figure 1:**
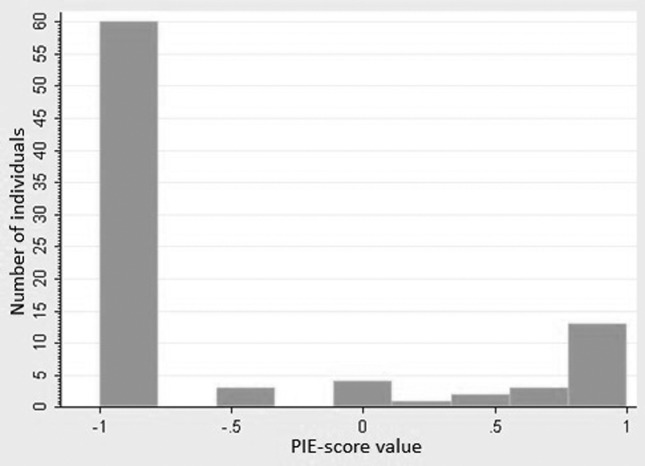
Histogram of distribution of iris color variation. X axis: PIE-score (Pixel Index of the Eye) ranged from -1 to 1 (i.e. from brown to blue, respectively). Y axis: Number of individuals with each PIE-score value.

We observed a non normal distribution of iris color with major prevalence of brown (Figure 1). The vast majority of DIAT results were consistent with the expected PIE-score (80.2% agreement for the whole sample). Nevertheless, not all results from DIAT could be included in our statistical analysis because of non-concordance of some PIE-scores with visual categorization of irises. Errors were mainly present in the group of irises visually considered as brown (20.9%), with lower influence in the group visually considered as blue or green (14.7%).

Table 1 shows the genotype frequencies that were obtained. The markers fitted HWE for the whole sample. When analyzed separately by iris color, *HERC2* rs12913832 did not fit HWE within the group of irises visually determined as brown eyes (LR=8.429; *p*=0.004), while *TYR* rs1373350 also showed a tendency to HW disequilibrium within the blue group (LR=5.091; *p*=0.078).

**Table 1:** Genotypic frequencies for five markers related to eye color. n: sample size.

Marker	Genotype	Blue eyes	Brown eyes	Population frequency
rs12913832	AA	0.0000 ± 0.0000	0.47826 ± 0.05207	0.38261 ± 0.04532
n=115	AG	0.17391 ± 0.07903	0.48913 ± 0.05212	0.42609 ± 0.04611
	GG	0.82608 ± 0.07903	0.03261 ± 0.01851	0.19130 ± 0.03668
rs12203592	CC	0.78261 ± 0.08601	0.84783 ± 0.03745	0.83478 ± 0.03463
n=115	CT	0.17391 ± 0.07903	0.13043 ± 0.03511	0.13913 ± 0.03227
	TT	0.04348 ± 0.04252	0.02174 ± 0.01520	0.02609 ± 0.01486
rs12896399	GG	0.47619 ± 0.10899	0.46914 ± 0.05545	0.47059 ± 0.04942
n=102	GT	0.28571 ± 0.09858	0.41975 ± 0.05484	0.39216 ± 0.048342
	TT	0.23810 ± 0.09294	0.11111 ± 0.03492	0.137250.03407
rs16891982	CC	0.00000 ± 0.00000	0.04396 ± 0.02149	0.03509 ± 0.01723
n=114	CG	0.30435 ± 0.09594	0.32967 ± 0.04928	0.32456 ± 0.04385
	GG	0.69565 ± 0.09594	0.62637 ± 0.05071	0.64035 ± 0.04495
rs1393350	AA	0.08696 ± 0.05875	0.07692 ± 0.02793	0.07895 ± 0.02526
n=114	AG	0.47826 ± 0.10416	0.26374 ± 0.04619	0.30702 ± 0.04320
	GG	0.43478 ± 0.10337	0.65934 ± 0.04968	0.61403 ± 0.04560

Nucleotide diversity for the five SNPs was 0.3306 ± 0.2287; the mean observed heterozygosity was 0.3178 ± 0.0758, and the mean expected heterozygosity was 0.3335 ± 0.1277. *Fis* values were under 15% and non-significant, with rs12203592 in the limit of significance (*Fis*=19%; *p*=0.06264 ± 0.00025), which could be explained by the low frequency of the T allele for this marker.

When calculating genotype-phenotype association by a median regression, after discarding the underrepresented genotypes (Table 2), we only found a significant association of *HERC2* rs12913832 GG with blue iris color (*p* < 0.01). No association with iris color was found for the other markers.

**Table 2:** Results from median regression. REF: reference variable, sd: standard deviation, IC95: 95% confidence interval. *: removed from the analysis for being unrepresentative.

Marker	Genotype	Coefficient	sd	P-value	IC95
rs12913832	AA	REF	REF	REF	REF
	AG	0.02	0.08	0.77	[(-0.13) – 0.18]
	GG	1.82	0.1	< 0.01	[1.62 – 2.01]
rs12203592	CC	REF	REF	REF	REF
	CT	0.03	0.1	0.8	[(-0.17) – 0.23]
	TT	*	*	*	*
rs12896399	GG	REF	REF	REF	REF
	GT	-0.02	0.08	0.83	[(-0.17) – 0.14]
	TT	0.04	0.11	0.7	[(-0.17) – 0.26]
rs16891982	CC	*	*	*	*
	CG	0.02	0.08	0.83	[(-0.14) – 0.18]
	GG	REF	REF	REF	REF
rs1393350	AA	0	0.13	0.98	[(-0.26) – 0.25]
	AG	0.01	0.08	0.85	[(-0.14) – 0.17]
	GG	REF	REF	REF	REF

Considering the sex distribution, the Chi-square test with Yates correction was non-significant (0.811 with *p*=0.367), leading to conclude that for this sample size it was not possible to confirm an association between sex and iris color as has been previously proposed (Martinez-Cadenas *et al.*, 2013).

According to the *Fst* results (Table 3) and after applying Bonferroni correction, the Buenos Aires population should be considered as different from the other populations of the world for the analyzed polymorphisms, being most distant from the African populations and closest to the Native American ones here included for comparison. The AMOVA values were not significant (data not shown).

**Table 3:** Fst values obtained from interpopulation comparison. *p=0.00000. All values are significative, except for **p=0.01802 ± 0.0121.

	Buenos Aires	Africa	America	Europe	East Asia
Buenos Aires	0				
Africa	0.28325*	0			
America	0.11023*	0.17090*	0		
Europe	0.20880*	0.52826*	0.38470*	0	
East Asia	0.21005*	0.15482*	0.02330**	0.50612*	0

**Figure 2:**
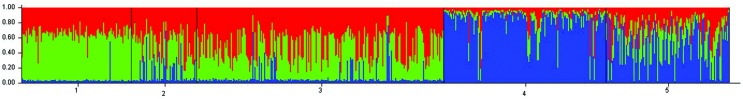
Structure analysis of different populations: 1-African, 2-Native American, 3-East Asian, 4-European, 5-Buenos Aires. Each color represents an assumed ancestral population. Each individual is represented by a single vertical line divided into K segments of different colors.

A population structure analysis was made assuming mixed ancestry. Structure Harvester (Earl and vonHoldt, 2012) was used to estimate the most probable K (Table 4). The bar plot grouped by population for K=3 shown in Figure 2 presented an evident differentiation of the Buenos Aires population from African, Native American, East Asian, and European populations.

**Table 4:** Most probable K (in bold letter) of the Structure analysis shown above. Performed with Structure Harvester

K	Reps	Mean LnP(K)	Stdev LnP(K)	Ln’(K)	Ln’’(K)	Delta K
2	20	-2198.195000	28.852738	—	—	—
**3**	**20**	**-2404.380000**	**175.883953**	**-206.185000**	**340.710000**	**1.937130**
4	20	-2269.855000	136.131942	134.525000	87.505000	0.642795
5	20	-2222.835000	114.639555	47.020000	—	—

**Figure 3:**
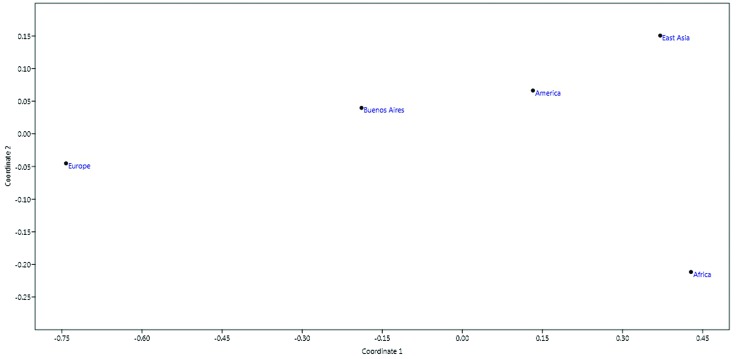
MDS matrix performed with Past. The distance among populations is calculated from Fst. Stress value=0.

The MDS matrix put the Buenos Aires population at similar distances from Europeans and East Asians, closer to Native Americans, and less related to African populations (Figure 3). Stress value analysis performed with PAST resulted in 0. For confirmation of this value the analysis was rerun using SSPS software, and this resulted in 0.00788.

## Discussion

Eye color is a polygenic trait and requires the analysis of multiple genes for phenotype assignment in forensic practice or for other purposes. In this study we present data on the phenotypic variation of iris and the genetic variation of five polymorphisms related to eye color, localized in different genes, and we assessed the reliability of applying the methods for eye color prediction in individuals from Buenos Aires with the same criteria as those used in other populations of the world. At present, these polymorphisms and genotype-phenotype relationship have not been studied yet in our population.

Good quality images and direct observation of iris color agreed with the PIE-scores resulting from DIAT. According to Andersen *et al.* (2013), the effect of light reflection in dark-brown eyes is more likely to confound the analysis (showing values near 0) than in blue eyes. Hence, those images were not included in the statistical analysis. In our sample, as in the Latin American population in general, the majority of the individuals are brown-eyed. Therefore, the use of this software in our population needs careful attention to avoid this problem, by reducing to minimum the iris light incidence when the photograph is taken.

As reported by Andersen *et al.* (2013), the software does not accurately distinguish among different tonalities, and not only images of dark and light-brown eyes, but also images of dark and light-blue irises might result in similar PIE-scores.

The HW disequilibrium seen for rs12913832 can be explained by association of homozygous genotype GG with blue iris color, resulting in a lower observed proportion of this genotype among brown-eyed individuals. This is in accordance with reports for this SNP as one of the most important markers for determination of iris color in the great majority of populations (Liu *et al.*, 2009; Mengel-From *et al.*, 2009; Pospiech *et al.*, 2011; Walsh *et al.*, 2012; Kastelic *et al.*, 2013; Ruiz *et al.*, 2013; Wilde *et al.*, 2014).

As expected, the relationship genotype-phenotype for rs12913832 was the same as that observed in other populations (Sulem *et al.*, 2007; Eiberg *et al.*, 2008; Sturm *et al.*, 2008; Nan *et al.*, 2009; Liu *et al.*, 2010; Valenzuela *et al.*, 2010; Spichenok *et al.*, 2011; Sturm and Duffy, 2012; Walsh *et al.*, 2011a,b, 2012; Kastelic and Drobnic, 2012; Beleza *et al.*, 2013; Draus-Barini *et al.*, 2013; Hart *et al.*, 2013; Kastelic *et al.*, 2013; Lim and Oh, 2013; Ruiz *et al.*, 2013; Wilde *et al.*, 2014). The G allele is associated to blue iris color, and the A allele to brown color. The G allele has been reported as a consequence of a founder mutation in *HERC2* (SNP rs12913832) which emerged at the East or Northwest of the Black Sea, about 6,000 to 10,000 years ago (Cavalli-Sforza and Feldman, 2003; Eiberg *et al,.* 2008) and extended along European populations due to sexual selection (Cavalli-Sforza and Feldman, 2003; Eiberg *et al.*, 2008; Rodrigues Nogueira Forti and Young, 2016). The blue-eye associated homozygote rs12913832-GG genotype, as well as the heterozygote genotype, are much more frequent in Europe (G frequency=0.79 according to Beleza *et al.* (2013), and 0.71 according to Wilde *et al.* (2014) in the surrounding areas, such as the Middle East and West Asia, where blue and intermediate eye colors are expected. In contrast, populations of African origin totally lack this allele. On the other hand, the brown eye associated the rs12913832-AA genotype is present everywhere across the continents, and it is nearly the only one found in certain areas of the world where no blue eye color is expected (Walsh *et al.*, 2011a). In the Buenos Aires population, the AA genotype is more frequent than in Europe, in accordance with a differential transoceanic migratory contribution to the native genetic background of our population (Avena *et al.*, 2006), and our results agreed with the varied ethnic composition of our sample. With regard to migratory processes, the presence of rs12913832-G allele can be attributed to migrations and, as a consequence, blue and green eyes in the Argentinian population, since these iris colors have the major occurrence among Europeans and individuals descending from them (Sturm and Frudakis, 2004). However, the migratory component coming from Europe was mainly from regions with a lower proportion of blue eyes (Spain and Italy), compared to whole Europeans (Devoto, 2007). Additionally, populations from neighboring countries also contributed with brown-eyed individuals to the Buenos Aires population.

The other markers fit HWE, and *Fis* values were non-significant, suggesting that important genetic drift, selection or inbreeding processes are not occurring at present in the population under study.

As the Buenos Aires population has a certain Native American contribution (Avena *et al.*, 2012), we searched in our sample for the presence of male Native American markers, such as M3 (Underhill *et al.*, 1996), M242 and M346 in the Y-chromosome, following Jurado-Medina *et al.* (2014). We only found 2% of M3 (1 brown-eyed man among 48 men), a result less common than the frequency observed in previous data (4%) (Martínez Marignac *et al.*, 2004) although still present. In this work, we did not perform an analysis of individual mitochondrial ancestry, which could result in a higher percentage of a Native component, as previously reported (Corach *et al.*, 2010). Further research focused on Native American contribution to ancestry and genetic association with iris color could reveal whether other mutations are influencing these results.

In this work, an association with iris color was not detected for four markers, contrary to previous reports from other authors (Sulem *et al,.* 2007; Liu *et al.*, 2009; Valenzuela *et al.*, 2010; Spichenok *et al.*, 2011; Walsh *et al.*, 2011a; Kastelic and Drobnic, 2012; Hart *et al.*, 2013; Lim and Oh, 2013; Ruiz *et al,.* 2013; Fernandes Durso *et al.*, 2014; Wilde *et al.*, 2014). In the case of the SNP rs12203592 from *IRF4* gene, the reduced frequency observed for the T allele might interfere in the possibility of finding an association with iris color. Another factor might be the sample size, and we also have to consider that, in the population under study, the number of individuals with blue eyes is much lower than individuals with brown eyes. It is important to note that iris color depends, in part, on light scattering and absorptive properties of extracellular components, especially in lightly pigmented irises (Eagle, 1994), so this fact can be a complication for defining a clear relationship between each polymorphism and iris color.

In contrast to other studies (Martinez-Cadenas, 2013, 2014), it was not possible to demonstrate an association between sex and iris color in our population. Our results were consistent with the lack of association, as it was proposed by Liu *et al.* (2014).

Results obtained for *Fst* consistently showed that the Buenos Aires population is genotypically different from other populations of the world, reaffirming the importance of studying these genes in our population for people identification based on iris color. The structure analysis also pointed out this differentiation. The results for the Buenos Aires population were more similar to native populations from other American countries, and in second place, to European populations, suggesting a considerable contribution of Native Americans to the current genetic composition of the Buenos Aires population. However, the percentage of Native contribution in this province is much lower than in other regions of the country (Marino *et al.*, 2007, Avena *et al.*, 2012, Motti *et al.*, 2013). Hence, these results, which are in contrast with values of the demographic composition (2.5% of Native American descendants for the whole country, according to INDEC, 2010), could be explained by recent migration from neighboring countries (Avena *et al.*, 2006) searching for work opportunities (Oyhenart *et al.*, 2013). Moreover, it has been reported that a considerable proportion of the Argentinian population has at least one Indigenous American ancestor, in contrast to the perception of most Argentine people, who identify themselves as of European-descent, with only 1% of the total population self-identifying as descendants of an indigenous group (Avena *et al.*, 2012).

According to our results, Africans were the most distant from Native American, European, East Asian and Buenos Aires populations, in agreement with the age of divergence of African populations from the rest of the world (Cavalli-Sforza and Feldman, 2003; Liu *et al.*, 2006; Reyes-Centeno *et al.*, 2015). Similarities between Asians and Native Americans could be explained by an ancient common origin of both populations (Cavalli-Sforza and Feldman, 2003) that share an absence of blue eyes (Beals and Hoijer, 1965).

Our results suggest that iris color SNPs should be analyzed for the population in which the system IrisPlex is going to be implemented. Another option is to select the SNPs to use in identification after an evaluation of the population under consideration. The differentiation found among populations is consistent with the fact that these markers are related to a phenotype with an important regional adaptive value.

We conclude that the genetic background for iris color in the Buenos Aires population is genetically different to that of other countries. The differences we found might be due to the admixed ethnic composition of Argentina. Buenos Aires presents a population identity that differentiates it from other populations of the world, and for that reason, the methods of analysis used in European populations should be carefully applied in this case. Our results emphasize the importance of analyzing the variation of these genes and their relationship with iris color, not only in this province, but along the Argentinian country for people identification based on iris color.
